# Multi-source joint domain adaptation for cross-subject and cross-session emotion recognition from electroencephalography

**DOI:** 10.3389/fnhum.2022.921346

**Published:** 2022-09-15

**Authors:** Shengjin Liang, Lei Su, Yunfa Fu, Liping Wu

**Affiliations:** School of Information Engineering and Automation, Kunming University of Science and Technology, Kunming, China

**Keywords:** affective brain–computer interface, affective computing, EEG, emotion recognition, domain adaptation

## Abstract

As an important component to promote the development of affective brain–computer interfaces, the study of emotion recognition based on electroencephalography (EEG) has encountered a difficult challenge; the distribution of EEG data changes among different subjects and at different time periods. Domain adaptation methods can effectively alleviate the generalization problem of EEG emotion recognition models. However, most of them treat multiple source domains, with significantly different distributions, as one single source domain, and only adapt the cross-domain marginal distribution while ignoring the joint distribution difference between the domains. To gain the advantages of multiple source distributions, and better match the distributions of the source and target domains, this paper proposes a novel multi-source joint domain adaptation (MSJDA) network. We first map all domains to a shared feature space and then align the joint distributions of the further extracted private representations and the corresponding classification predictions for each pair of source and target domains. Extensive cross-subject and cross-session experiments on the benchmark dataset, SEED, demonstrate the effectiveness of the proposed model, where more significant classification results are obtained on the more difficult cross-subject emotion recognition task.

## Introduction

Affective computing is emotion-related computing that helps improve the user experience during a human-computer interaction (Picard, 2000; Tao and Tan, 2005). As one of the basic tasks of affective computing, emotion recognition can generate labels that correspond to emotions such as positive, neutral, or negative emotions (Zheng and Lu, 2015; Cambria et al., 2017). Emotion plays an important role in rational human behavior (Picard, 2000) and influences people’s daily lives. For example, a positive emotion often makes people feel good, while a negative emotion may have the opposite effect. Therefore, research on emotions can help people better cope with the impact of different emotions to improve their quality of life. Facial expression and voice are two recognized forms of sentic modulation that can be utilized to identify human emotions (Picard, 2000). However, externally presented behaviors, such as facial expression and voice, are strongly influenced by individual factors. In addition, they are very easy to disguise, for instance, one may pretend to smile in some situations and hide an unusual voice in others. To achieve more natural emotional communication in the process of human-computer interaction, we need to find more reliable methods for emotion recognition.

In recent years, many researchers have gradually focused on the interesting field of brain–computer interfaces. By using neural activity generated by the brain, the brain–computer interface enables harmonious interactions between the user and the computer (Vallabhaneni et al., 2005). This makes sense for some people, such as those with muscle movement difficulties (Vallabhaneni et al., 2005), who now have an opportunity to apply a brain–computer interface to communicate more amicably with devices or other people. An affective brain–computer interface (Nijboer et al., 2009) is a technique that can detect the user’s emotional states from brain activity (Zheng and Lu, 2015). There are various methods of recording brain activity (Ramadan and Vasilakos, 2017), including electrocorticography (ECoG), magnetoencephalography (MEG), and electroencephalography (EEG). EEG is widely used in the study of affective brain–computer interfaces (Zheng and Lu, 2015; Chai et al., 2016; Zhang et al., 2021) due to its many advantages, such as non-invasiveness, practicality, high temporal resolution, and high accuracy (Vallabhaneni et al., 2005; Ramadan and Vasilakos, 2017; Egger et al., 2019). Unlike external modalities such as facial expression and voice, EEG signals are not easily camouflaged, and thus EEG-based emotion recognition methods can more objectively evaluate the user’s emotional states.

However, the brain presents non-stationary states under the interference of some internal or external factors (Ramadan and Vasilakos, 2017), which means that an EEG measuring brain activity has the property of non-stationarity (Paluš, 1996). Hence, EEG signals vary within and between subjects, which poses a huge challenge to the research of affective brain–computer interfaces based on EEG. Personality, environment, and many other factors may cause individual differences in the EEG signals of different subjects. Furthermore, one’s EEG signals will constantly change over time. Existing studies have shown that there are large differences in the EEG data distributions of different subjects, and the EEG data distribution of the same subject varies at different time periods (Chai et al., 2016; Li et al., 2019b; Shen and Lin, 2019). As a result, the performance of the pretrained emotion recognition model is worse than expected when it is applied to other subjects or different sessions of the same subject, corresponding to cross-subject and cross-session scenarios, respectively. To this end, traditional methods (Zheng and Lu, 2015; Alhagry et al., 2017; Zhong et al., 2020) need to collect and label a large amount of EEG data for training a model from the subject waiting to be tested to satisfy the important assumption that training data and test data are independently and identically distributed (i.i.d.) in traditional machine learning. However, such methods undoubtedly will critically affect the user experience.

To decrease the amount of training data required for test subjects and reduce the time-consuming and laborious data labeling work, transfer learning (Pan and Yang, 2009) or domain adaptation (Patel et al., 2015) was introduced into EEG emotion recognition research to address cross-domain problems. Domain adaptation techniques can leverage the useful knowledge learned from existing subjects or sessions (referred to as the source domain) to facilitate learning on other subjects or sessions (referred to as the target domain). Many current domain adaptation methods tend to reduce the marginal distribution difference between the source and target domains while usually simply assuming that the conditional distributions between them will automatically match or remain unchanged. Moreover, most works simply merge multiple subjects from existing source domains into one single source domain, without considering the differences in data distribution among the different subjects. This may bring some performance gains to the model due to the increased amount of data. However, directly merging multiple source domains with different data distributions may cause confusion in the distribution of the merged data, which limits the further improvement of model performance (Zhu et al., 2019; Chen et al., 2021).

To take full advantage of the data distributions of multiple source domains, this paper proposes a novel multi-source joint domain adaptation (MSJDA) network to improve the generalization ability of the EEG emotion recognition model across subjects and sessions. We assume that the EEG data in the source domains are fully labeled, and the target domain data are unlabeled, which is congruent with unsupervised domain adaptation. We construct a multi-source EEG emotion recognition network, which is divided into three parts, a domain-shared feature extractor, domain-private feature extractors, and domain-private label predictors. First, a domain-shared feature extractor extracts the general features for all the domains. Second, each pair of source and target domains is further mined by a domain-private feature extractor for specific features that are beneficial for distinguishing emotion categories. Third, with labeled source domain data, a domain-private label predictor can be trained separately for each source domain. Moreover, we employ joint maximum mean discrepancy (JMMD) (Long et al., 2017) to match the joint distributions of each pair of source and target domains across multiple specific network layers. Our model can simultaneously train label predictors and reduce the distribution differences across domains. Finally, the predictions of target domain samples can be jointly determined by the predictions of all the source classifiers.

There are two main contributions in this paper. The first is a multi-source joint domain adaptation network proposal, which helps to solve the problem of cross-domain generalization in EEG emotion recognition. The second is our extensive cross-subject and cross-session transfer experiments on a publicly available emotion EEG dataset that verify the effectiveness of the proposed method.

The remainder of this paper is organized as follows. Section “Related work” presents some of the research most relevant to this paper. The proposed network model is described in detail in the section “Materials and methods.” The content related to the experiments, such as the dataset and the experimental results, will be given in the section “Experiments.” “Discussion” discusses and analyzes the proposed model. Finally, the section “Conclusion” summarizes our main work.

## Related work

Domain adaptation, especially in unsupervised scenarios of transferring knowledge from a labeled source domain to an unlabeled target domain, has been extensively studied in fields such as computer vision and natural language processing. In this section, we mainly review the domain adaptation algorithms that are most relevant to this paper. To address the challenge of model performance degradation across different subjects or sessions, domain adaptation techniques have been gradually applied to EEG emotion recognition in recent years. Among the numerous previous works, there are two popular types of approaches.

One approach employs metrics to measure and minimize the distribution discrepancy across domains. Once the cross-domain distance is reduced to an acceptable level, a model trained with source supervision can better predict target samples. One of the most commonly used metrics is the maximum mean discrepancy (MMD) (Gretton et al., 2006), which represents the distance between two distributions in a reproducing kernel Hilbert space (RKHS). Zheng et al. (2015) introduced transfer component analysis (TCA) (Pan et al., 2010) to address the cross-subject generalization problem in EEG emotion recognition. TCA can minimize the MMD between the source and target domains in a latent space to reduce the marginal distribution difference between them while preserving their useful data properties. Based on TCA, joint distribution adaptation (JDA) (Long et al., 2013) takes into account the conditional distribution by iteratively optimizing the pseudo labels of the target samples. Previous studies have shown that deep learning has the capability to automatically learn better feature representations, and thus there is a benefit to embedding domain adaptation into neural networks. A deep adaptation network (DAN) (Long et al., 2015) was applied to the problem of individual differences in EEG signals, where the multi-kernel MMD was calculated for the difference between source and target subjects in the last two layers of the neural network (Li et al., 2018). To avoid the degradation of the emotion classification model in cross-subject and cross-session situations, Liu et al. (2021) adopted joint adaptation networks (JAN) (Long et al., 2017) to align the joint distributions of two domains. However, they directly merged all subjects or sessions of the source domains into one single source domain without bearing in mind the multi-source scenario in practical application.

The other approach is to extract domain-invariant features through adversarial learning. Generative adversarial nets (GANs) (Goodfellow et al., 2014) generate new samples from random noise that are indistinguishable from the training data, meaning they can make two different distributions similar. Naturally, the idea of adversarial learning is introduced into the study of domain adaptation. Jin et al. (2017) exploited a domain-adversarial neural network (DANN) (Ganin and Lempitsky, 2015; Ganin et al., 2016) to improve the accuracy of emotion recognition across subjects. The gradient reversal layer (GRL) included in DANN is a key component to extract the domain-invariant features. Through two stages of pretraining and adversarial training, Wasserstein generative adversarial network domain adaptation (WGANDA) (Luo et al., 2018) learned independent source and target generators to map the corresponding domain to a common feature space, in which the target distribution was moved closer to the source distribution to deal with the cross-subject domain shift problem. Li et al. (2019a) matched the marginal and conditional distributions of latent representations from the source and target domains in the shallow and deep layers of the network, respectively. Although their method approximated matching joint distributions, they only considered the scenario of one single source domain. To reduce the calibration time of the cross-subject emotion recognition model, Zhao et al. (2021) explicitly extracted the shared representations of all subjects and the private representations of each subject and enhanced the domain invariance of the shared representations through a domain classifier. They treated each subject independently, but their approach only considered the marginal distributions between domains.

The above methods validated their performance under different settings, indicating that domain adaptation can generalize models for EEG emotion recognition. In affective brain–computer interfaces, we are usually given labeled data from multiple subjects or sessions. Moreover, the data distributions of the different subjects differ from each other, as do the different sessions of the same subject. However, most of the above methods simply merge different subjects or sessions of the source domains into one single domain, ignoring the differences between them. Many methods assume that after aligning the marginal distributions of source and target domains, their conditional distributions are automatically aligned or remain unchanged throughout the process, which may not always be the case in practical application. Therefore, this paper considers the cross-subject and cross-session scenarios and treats different source subjects or sessions as different source domains, while the target domain contains one subject or one session. We then propose a multi-source joint domain adaptation network that aligns the joint distribution of the deep features and classification predictions for each source domain and target domain.

## Materials and methods

This section introduces a metric for the difference between two joint distributions, followed by a formal description of our proposed multi-source joint domain adaptation network.

### Metric between joint distributions

In the domain adaptation problem, if the data distributions of two domains are different, the intuitive approach is to seek some well-performing metrics to help reduce the domain differences. Related studies (Gretton et al., 2006; Song et al., 2013; Long et al., 2017) reveal that a probability distribution can be embedded as a point in an RKHS *ℋ* by an implicit feature map ϕ, which is called the kernel embedding of distribution. One of its most common applications is MMD (Gretton et al., 2006), which is able to determine whether two samples come from different distributions. MMD can be expressed as the distance between the kernel embeddings of the corresponding marginal distributions. In actual computation, the implicit feature map ϕ is usually implemented by operations on kernel functions, such as the Gaussian RBF kernel and Laplace kernel (Song et al., 2013).

When two or more random variables are involved, according to Song et al. (2013) and Long et al. (2017), the kernel embedding of their joint distribution can be represented as an element in a tensor product feature space. Specifically, for the joint distribution *P*(^**Z**1^,…,^**Z***m*^) obeyed by *m* variables ^**Z**1^,…,^**Z***m*^, a set $D_{Z^{1}\,\ldots \,Z^{m}}={\left\{\left({\text{z}}_{i}^{1},\ldots ,{\text{z}}_{i}^{m}\right)\right\}}_{i=1}^{n}$ of *n* i.i.d. samples is given. Then, using an implicit feature map ${⊗}_{l=1}^{m}\phi^{l}$ in the tensor product feature space ${⊗}_{l=1}^{m}{ℋ}^{l}$, the kernel embedding of this joint distribution can be estimated empirically as:


(1) $$\begin{array}{c} {𝒞}_{Z^{1}\,\ldots \,Z^{m}}=\frac{1}{n}\,\sum_{i=1}^{n}{⊗}_{l=1}^{m}\phi^{l}\,\left({\text{z}}_{i}^{l}\right). \end{array}$$

To measure the discrepancy between two joint distributions, Long et al. (2017) proposed a metric called the JMMD. The JMMD can be interpreted as the squared distance between the kernel embeddings of two joint distributions. It is often embedded in a neural network to evaluate the difference in the joint distributions of activations from two domains across multiple layers. Given two different domains sampled from joint distributions ^*Ps*^(^**X***s*^,^**Y***s*^) and ^*Pt*^(^**X***t*^,^**Y***t*^), their corresponding sample sizes are *n_s* and *n_t*, respectively. After forward propagation in the neural network, these two domains separately produce activations ${\left\{\left({\text{z}}_{i}^{s\,1},\ldots ,{\text{z}}_{i}^{s\,\left|ℒ\right|}\right)\right\}}_{i=1}^{n_{s}}$ and ${\left\{\left({\text{z}}_{j}^{t\,1},\ldots ,{\text{z}}_{j}^{t\,\left|ℒ\right|}\right)\right\}}_{j=1}^{n_{t}}$ in multiple domain-specific layers *ℒ*. Their joint distributions ^*Ps*^(^**Z***s*1^,…,^**Z***s*|*ℒ*|^) and ^*Pt*^(^**Z***t*1^,…,^**Z***t*|*ℒ*|^) are considered to still be different and can act as proxies for the joint distributions ^*Ps*^(^**X***s*^,^**Y***s*^) and ^*Pt*^(^**X***t*^,^**Y***t*^) of the original data, respectively (Long et al., 2017). Based on the empirical kernel embedding of the joint distribution mentioned above, the finite sample estimate of the JMMD between the two joint distributions ^*Ps*^(^**Z***s*1^,…,^**Z***s*|*ℒ*|^) and ^*Pt*^(^**Z***t*1^,…,^**Z***t*|*ℒ*|^) is


(2) $$\begin{array}{c} J\,M\,M\,D\,\left(P^{s},P^{t}\right)\\ ={||\frac{1}{n_{s}}\,\sum_{i=1}^{n_{s}}{⊗}_{l=1}^{\left|ℒ\right|}\phi^{l}\,\left({\text{z}}_{i}^{s\,l}\right)-\frac{1}{n_{t}}\,\sum_{j=1}^{n_{t}}{⊗}_{l=1}^{\left|ℒ\right|}\phi^{l}\,\left({\text{z}}_{j}^{t\,l}\right)||}_{{⊗}_{l=1}^{\left|ℒ\right|}{ℋ}^{l}}^{2}. \end{array}$$

### The multi-source joint domain adaptation network

In the cross-domain EEG emotion recognition problem, it is often necessary to deal with multiple domains with significantly different distributions. To reduce the cost of labeling training data for the target task, experience can be gained from multiple labeled source subjects to help predict the target subject. When data are prone to staleness, the predictions of a new session for one subject can benefit from its known multiple labeled sessions.

Unlike many existing studies that directly combine multiple source domains into one single source domain, we try to explore a more challenging and practical multi-source scenario in which each source domain with a different distribution is treated as an independent source domain. In multi-source unsupervised domain adaptation, *K* distinct source domains *D*_*s*_={_*Ds*__*k*_|*k* = 1,…,*K*}, where the *k*-th source domain $D_{s_{k}}={\left\{\left({\text{x}}_{i}^{s_{k}},{\text{y}}_{i}^{s_{k}}\right)\right\}}_{i=1}^{n_{k}}$ contains *n_k* i.i.d. labeled examples drawn from the joint distribution ^*Ps*_*k*_^(^**X***s*_*k*_^,^**Y***s*_*k*_^), and a target domain $D_{t}={\left\{{\text{x}}_{j}^{t}\right\}}_{j=1}^{n_{t}}$ of *n_t* i.i.d. unlabeled examples sampled from the joint distribution ^*Pt*^(^**X***t*^,^**Y***t*^), are generally given. It is assumed that the joint distributions of different source domains are not equal to each other, and each source domain and target domain also have different joint distributions. Our aim is to reduce the difference between the labeled *K* source domains and the unlabeled target domain to make the extracted features domain-invariant so that the source learner has better performance when applied to the target domain.

#### Network framework

**FIGURE 1:**
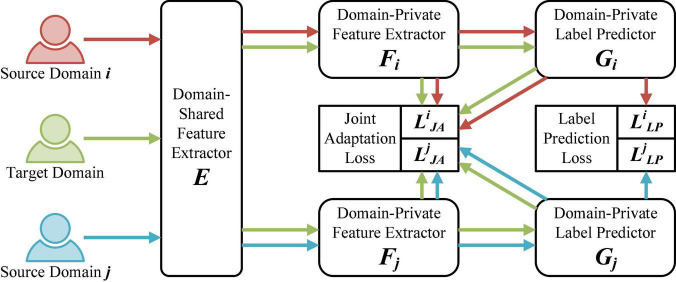
The proposed multi-source joint domain adaptation (MSJDA) framework. For brevity, here, only the **i**-th and **j**-th (**i**≠**j** and **i**,**j** ∈ {**1**,…,**K**}) of the **K** source domains are selected to present our network framework.

We propose a network capable of handling multiple domains with different distributions for the cross-domain EEG emotion recognition problem. As shown in Figure 1, our proposed Multi-Source Joint Domain Adaptation (MSJDA) framework consists of three parts: (a) domain-shared feature extractors, (b) domain-private feature extractors, and (c) domain-private label predictors.

A domain-shared feature extractor *E* refers to a shared network structure through which the EEG data of all the source and target domains first pass. Its goal is to enable the neural network to learn abstract representations that are shared by different domains. These representations should be general to all domains for subsequent further processing. For an input sample **x** from a specific domain, the feature extracted by *E* is denoted as **e**=*E*(**x**).

Each of the domain-private feature extractors, *F* = {*F*_*k*_|*k* = 1,…,*K*}, is a separate feature extraction structure for each pair of the *k*-th source domain and the target domain, which follows the domain-shared feature extractor *E*. In other words, there are *K* network branches after *E*, each of which corresponds to one source domain. The role of *F_k* is to extract some deep representations relevant to the final classification task for each pair of source and target domains. If **e**_*k*_ is used to uniformly represent the general features of the *k*-th source domain or the target domain input to *F_k*, then the output corresponding to *F_k* is **f**_*k*_=*F*_*k*_(**e**_*k*_).

The domain-private label predictors are denoted as *G* = {*G*_*k*_|*k* = 1,…,*K*}, where *G_k* is connected to *F_k*. We treat each labeled source domain as an independent source domain. Each source domain has an independent label predictor, which is trained by means of supervised learning and connected to the corresponding private feature extractor. The output of the *k*-th label predictor can be expressed as **g**_*k*_=*G*_*k*_(**f**_*k*_).

Our work is inspired by the work of Zhu et al. (2019), who proposed a multiple feature spaces adaptation network to address the cross-domain image classification problem in computer vision. They utilized MMD to match the marginal distributions of each pair of source and target domains and reduced the prediction divergence of individual source classifiers on target samples. Different from the work of Zhu et al. (2019), our method separately considers the joint distributions of each pair of source and target domains and does not need to align classifiers from different source domains.

#### Model optimization

For a labeled source domain sample, the error between its predicted label and its ground-truth label, which we call the label prediction loss, can be calculated by the cross-entropy loss function *L*_*CE*_(⋅,⋅). The label prediction loss for the *k*-th source domain is:


(3) $$\begin{array}{c} L_{L\,P}^{k}=\frac{1}{n_{k}}\,\sum_{i=1}^{n_{k}}L_{C\,E}\,\left(G_{k}\,\left(F_{k}\,\left(E\,\left({\text{x}}_{i}^{s_{k}}\right)\right)\right),{\text{y}}_{i}^{s_{k}}\right). \end{array}$$

After passing through the domain-shared feature extractor, the unlabeled data of the target domain will enter the network branch of each source domain. In this way, we measure the JMMD between each source domain and target domain in a one-to-one manner. The network model proposed here only takes into account the case where each label predictor has one layer, but it can also be easily generalized to the multilayer case. The domain-private feature **f**_*k*_ and label prediction **g**_*k*_, i.e., the activations of the last two layers of each network branch, are used to compute the joint distribution difference (joint adaptation loss) in these layers for each pair of source and target domains. This difference is taken as an approximation of the difference in the original joint distributions of the corresponding source and target domains. Then, the joint adaptation loss between the *k*-th source and target domains is computed as:


(4) LJ⁢Ak=||1nk∑i=1nkϕ1(fk,isk)⊗ϕ2(gk,isk)-1nt∑j=1ntϕ1(f)k,jt⊗ϕ2(gk,jt)||ℋ1⊗ℋ22.

Therefore, the total loss function is the sum of the label prediction loss and the joint adaptation loss; that is, the objective function of the proposed network is


(5) $$\begin{array}{c} L_{t\,o\,t\,a\,l}=\frac{1}{K}\,\sum_{k=1}^{K}\left(L_{L\,P}^{k}+\lambda \,L_{J\,A}^{k}\right), \end{array}$$

where λ > 0 is the tradeoff parameter between the two losses. The objective function is optimized as


(6) $$\begin{array}{c} \min_{E,F,G}L_{t\,o\,t\,a\,l}, \end{array}$$

to improve the performance of the label predictors and reduce the distribution difference between the source and target domains, thereby learning discriminative and domain-invariant feature representations.

During training, the EEG data samples of one of the source domains and target domain are input to fit the model in each iteration. At test time, the prediction of each sample in the target domain is determined by the average of the outputs of all the label predictors.

## Experiments

We compare the proposed MSJDA network with several baseline methods in cross-subject and cross-session scenarios.

### Dataset

**FIGURE 2:**
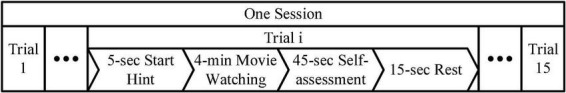
Flowchart of the acquisition of EEG in one session for one subject.

To verify the effectiveness of the proposed MSJDA model, we conducted extensive cross-domain EEG emotion recognition experiments on the benchmark dataset SEED (Zheng and Lu, 2015). SEED is a public dataset for evaluating the performance of EEG emotion recognition models, in which the EEG signals were collected from 15 subjects with an average age of 23.27, consisting of 7 males and 8 females. When they watched some specially selected emotional movie clips, the electrode caps they wore synchronously recorded the corresponding EEG signals. The electrode cap used has 62 channels, and their positional layout follows the international 10-20 system. Each subject was asked to participate in three experiments that collected EEG data on different days. Each such experiment was called a session. In a data collection session, each participant was required to perform 15 consecutive trials. In each trial, each subject was required to watch a carefully prepared emotional movie clip of approximately 4 min in length and self-evaluate it with the aim of eliciting positive, neutral, or negative emotions. The number of movie clips was the same for each emotion; in other words, each emotion corresponded to roughly the same number of samples. The flowchart of EEG acquisition is shown in Figure 2.

The raw EEG data first were preprocessed, including downsampling to 200 Hz, removing artifacts from the signals, and passing through a bandpass filter between 0 and 75 Hz. Next, the preprocessed data were further divided into five frequency bands, namely, delta (1–3 Hz), theta (4–7 Hz), alpha (8–13 Hz), beta (14–30 Hz), and gamma (31–50 Hz). In previous studies (Duan et al., 2013; Zheng and Lu, 2015), the differential entropy features were shown to be more suitable for emotion recognition than other features under the same conditions. Many domain adaptation works in EEG emotion recognition have adopted differential entropy features as sample data fed to the models (Zheng et al., 2015; Li et al., 2018; Liu et al., 2021; Zhao et al., 2021). So we follow them to use such features. The differential entropy feature (Duan et al., 2013) was extracted from the time series data of each frequency band for each channel as:


(7) $$\begin{array}{c} D\,E\,\left(X\right)=-\int_{X}p\,\left(x\right)\,\log\left(p\,\left(x\right)\right)\,dx=\frac{1}{2}\,\log\left(2\,\pi \,e\,\sigma^{2}\right), \end{array}$$

where *X* is a random variable with Gaussian distribution *N*(μ,σ^2^), and its corresponding probability density function is *p*(*x*). Finally, the number of samples per session was 3394. Each sample had a dimension of 310 (62 times 5) and was generated from one second of raw EEG data.

### Implementation details

We conducted the cross-domain transfer experiments in two scenarios. One scenario was the cross-subject transfer experiment, which takes the new subject as the target domain and the existing subjects as the source domains. The other was the cross-session transfer experiment that used the new session as the target domain and the existing sessions of the same subject as the source domains. In all the experiments, we adopted the leave-one-out cross strategy; that is, each time, one subject (session) was selected as the target domain, and the remaining subjects (sessions of the same subject) were selected as the source domains, so that each subject (session) had a chance to be the target domain. It should be noted that our MSJDA did not have access to the labels of the target domain during the training of the model, which means that it was an unsupervised domain adaptation method.

The proposed network was compared with several baseline methods. The regularization parameter *C* of the support vector machine (SVM) was fixed to 1, and a linear kernel was chosen for it. The settings of TCA (Zheng et al., 2015) and JDA (Long et al., 2013) were basically the same, that is, the dimension of the latent space was 30, the regularization parameter was 1, and a subset of size 5000 was randomly selected from all the source samples as the source domain data for the model training (it is difficult to include all the source samples with limited memory). In addition, since JDA is an iterative method, we chose its iteration number to be 10. Since TCA involves the process of dimensionality reduction, Kernel Principal Component Analysis (KPCA) (Schölkopf et al., 1997; Zheng et al., 2015), a traditional dimensionality reduction method, is often used for comparison. For KPCA, the number of components was set to 30, and the source domain contained the same number of samples as TCA. For the multilayer perceptron (MLP) and JAN (Liu et al., 2021), their entire architectures consist of multiple fully connected layers (512-256-128-64-32-3). The activations of the last two layers in the network were used to calculate the JMMD for JAN. The methods mentioned above combine all source domains into one single source domain. Methods without transfer techniques (SVM and MLP) directly apply their models trained on the source domain to the target domain.

For our proposed MSJDA method, the number of neurons in each layer of the domain-shared feature extractor is 512, 256, and 128. Each domain-private feature extractor has 2 layers with 64 and 32 neurons, respectively. Each domain-private label predictor has one layer with an output size of 3. Each source domain extends a network branch after passing through the domain-shared feature extractor, where the activations of the last two layers of each network branch are used to compute the joint adaptation loss.

Furthermore, Multi-Source EEG-Based Emotion Recognition Network (MEERNet) (Chen et al., 2021) is a multi-source method that computes MMD loss to reduce the difference between source and target domains, and its network structure is similar to our method.

For the neural network-based methods, the setting of the learning rate, α=α_0_/(1 + β*p*)^γ^, is the same as that in Ganin and Lempitsky (2015), where α_0_=0.0001, β=10, γ=0.75, and *p* (linearly increasing from 0 to 1) represents the training progress of the model. For JAN, MEERNet, and our MSJDA, the value of the tradeoff parameter is computed by λ=2/(1 + *e*^−η*p*^)−1 with η=10 so that it varies from 0 to 1, which also follows the setting in Ganin and Lempitsky (2015).

In addition, the differential entropy features for each session of each subject are separately standardized before being fed to the model to speed up convergence.

### Cross-subject experimental results

First of all, we verify the superiority of the proposed network in cross-subject experiments. Most of the existing studies only select the EEG data of one session from each subject for transfer experiments. For a more comprehensive comparison, we conducted cross-subject experiments in 3 different sessions. Specifically, each subject has EEG data from 3 sessions, and previous studies have shown that the data distribution varies between sessions. Accordingly, it is not appropriate to directly combine all sessions for each subject, so we conducted three cross-subject experiments. The data for the *i*-th (*i* ∈ {1,2,3}) cross-subject experiment consisted of the *i*-th session of all the subjects. The mean accuracy (Mean) of the *i*-th session refers to the average of 15 test accuracies obtained by taking each of the 15 subjects in turn as target domain (corresponding remaining subjects as source domains), where all subjects’ data are from the *i*-th session. The standard deviation (Std.) corresponding to the mean accuracy is also calculated.

**FIGURE 3:**
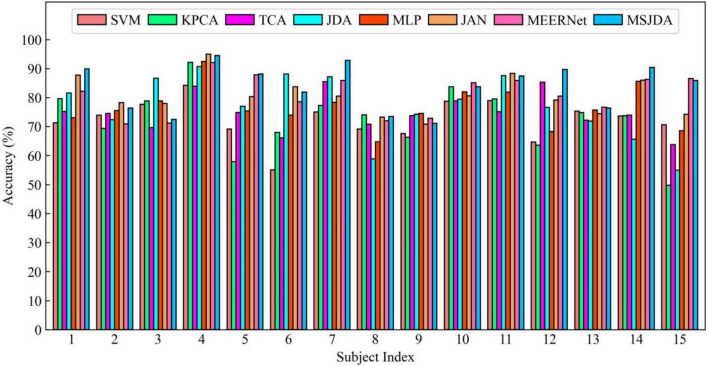
Results of cross-subject experiments in the first session.

**FIGURE 4:**
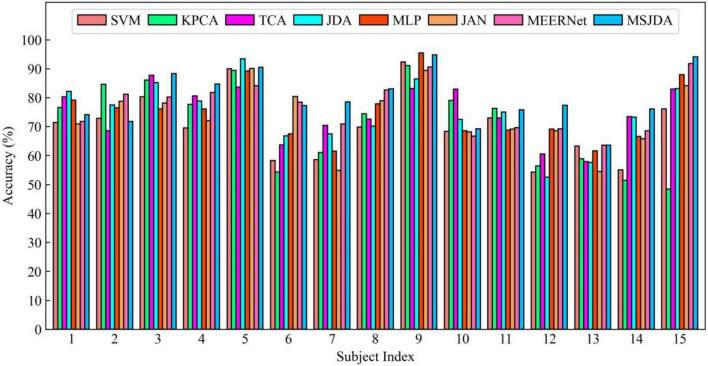
Results of cross-subject experiments in the second session.

**FIGURE 5:**
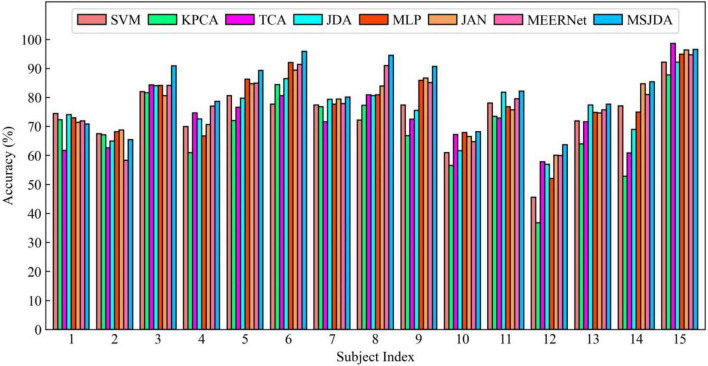
Results of cross-subject experiments in the third session.

The results of the cross-subject experiments are shown in Figures 3–5 and Table 1. “All Sessions” in Table 1 means that the experimental results of all three sessions were directly averaged. Some important observations can be drawn from the experimental results.

**TABLE 1:** Average results of cross-subject experiments (%).

Method	Session 1		Session 2		Session 3		All sessions		
	Mean	Std.	Mean	Std.	Mean	Std.	Mean	Std.	*P*-value
SVM	72.32	6.97	70.20	11.47	73.64	10.50	72.06	9.72	***
KPCA	72.57	10.57	71.04	14.55	68.68	13.28	70.76	12.72	***
TCA	74.89	6.38	74.75	9.23	72.96	10.59	74.20	8.75	***
JDA	76.84	10.84	74.81	10.95	75.72	9.53	75.79	10.26	***
MLP	76.59	7.06	74.79	10.06	77.05	11.00	76.15	9.36	***
JAN	80.68	6.55	73.58	10.74	78.22	9.74	77.49	9.45	***
MEERNet	80.96	6.87	76.74	8.75	78.48	11.02	78.73	9.01	***
MSJDA	83.60	7.82	79.95	9.12	81.97	11.15	81.84	9.37	–

The *p*-values correspond to paired samples *t*-tests between our proposed method and other comparison methods (***p < 0.0001).

First, methods based on deep neural networks perform much better than non-deep ones. The most obvious result is that the MLP (76.15%) without the transfer technique is better than the best non-deep method JDA (75.79%), which adopts the transfer technique. This indicates that, although the differential entropy features manually extracted from the raw EEG data are used in the experiments, a deep neural network can still discover some deep representations from such features that are beneficial for distinguishing emotion categories.

Second, among traditional methods without deep learning, the transfer learning-based methods (TCA and JDA) are better than SVM without transfer technique; the same finding is also found in the deep methods, that is, JAN, MEERNet, and MSJDA are clearly better than MLP. Therefore, transfer learning can reduce the difference between the source and target domains, making the model perform better in the target domain.

Third, both JDA and TCA are better than KPCA, which only performs dimensionality reduction. This shows that there is still a large difference between the source and target domains after only performing dimensionality reduction. By explicitly reducing the differences between domains, the distribution between domains can be better aligned, thereby improving the performance of the model on the target domain data.

Fourth, JDA with JDA performs better than TCA with only a marginal distribution adaptation. This shows that, compared to just adapting the marginal distributions of the source and target domains, adapting the joint distributions can reduce more differences between the domains, resulting in better results.

Fifth, our MSJDA outperformed JAN and MEERNet by 4.35% (*p* < 0.0001) and 3.11% (*p* < 0.0001) on average, respectively. Compared with JAN, such an observation suggests that in the multi-source scenario, the target domain acquires more beneficial knowledge from multiple independent source domains than from one single merged source domain. MSJDA narrows the distance between each source domain and target domain by aligning their joint distributions of deep features and predictions, making it better than MEERNet, which only considers single-layer feature adaptation to match marginal distributions. Hence, the superiority of the proposed MSJDA is demonstrated in the cross-subject experiments.

Sixth, we also note that in the cross-subject experiments in Session 2, most methods achieved worse results than the other sessions. It can be deduced that Session 2 should be more difficult to handle than the other two sessions. However, in Session 2, our method still achieved the highest mean accuracy, indicating that our model has better generalization abilities.

### Cross-session experimental results

For cross-session experiments, each subject needs to conduct three experiments, respectively, so that the three sessions of the same subject are taken as target domain in turn (the remaining sessions of the same subject as source domains). To better evaluate the performance of the model, we did not average the experimental results for each subject. Instead, the mean accuracy of the *i*-th (*i* ∈ {1,2,3}) session refers to the average of 15 test accuracies, each obtained by one of the 15 subjects using the *i*-th session as the target domain.

**FIGURE 6:**
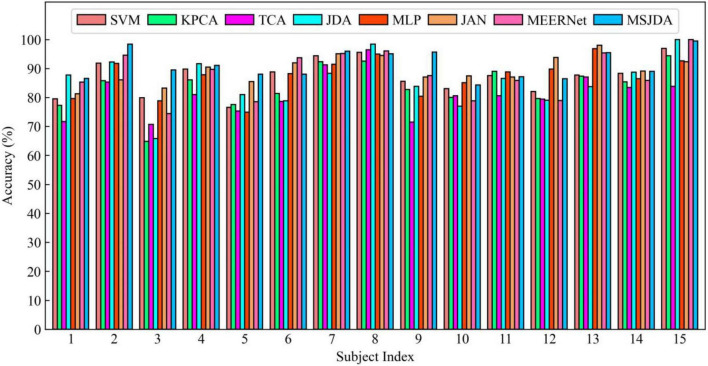
Results of cross-session experiments for each subject being transferred from their respective source sessions to their first session.

**FIGURE 7:**
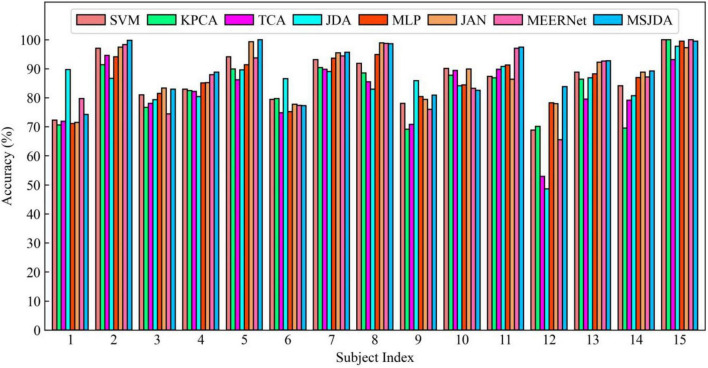
Results of cross-session experiments for each subject being transferred from their respective source sessions to their second session.

**FIGURE 8:**
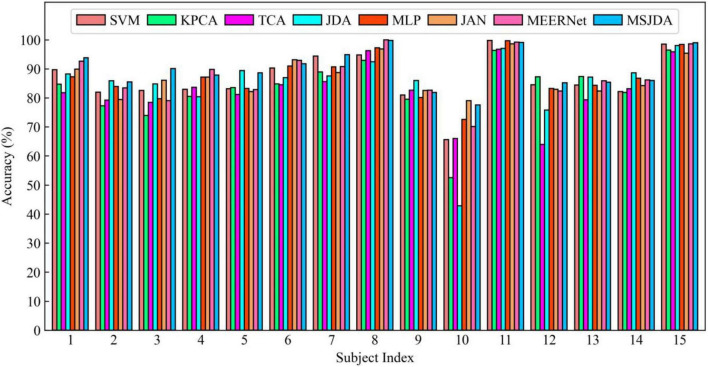
Results of cross-session experiments for each subject being transferred from their respective source sessions to their third session.

The results of the cross-session experiments are presented in Figures 6–8 and Table 2. “Session 1,” “Session 2,” and “Session 3” in Table 2 denote different target sessions (which are different from those in Table 1 for cross-subject experimental results), while “All Sessions” means that the results of all three cross-session experiments were directly averaged. Several observations can also be drawn from the cross-session experimental results.

**TABLE 2:** Average results of cross-session experiments (%).

Method	Session 1		Session 2		Session 3		All sessions		
	Mean	Std.	Mean	Std.	Mean	Std.	Mean	Std.	*P*-value
SVM	87.18	6.11	85.93	8.95	86.37	8.57	86.49	7.81	***
KPCA	83.75	7.54	82.62	9.57	83.19	10.67	83.19	9.15	***
TCA	81.11	7.27	81.18	10.81	82.55	9.40	81.61	9.08	***
JDA	85.53	8.69	83.92	10.84	84.72	12.84	84.72	10.69	**
MLP	87.18	6.33	86.38	8.01	87.01	7.46	86.86	7.14	***
JAN	89.53	4.72	88.05	8.80	87.21	6.33	88.26	6.74	*
MEERNet	87.98	7.79	87.07	10.57	87.76	8.25	87.60	8.76	**
MSJDA	91.36	4.87	89.54	8.83	89.75	6.58	90.22	6.84	–

The *p*-values correspond to paired samples *t*-tests between our proposed method and other comparison methods (*p < 0.01; **p < 0.001; ***p < 0.0001).

First, the results of the cross-session experiments are generally much better than those of cross-subject experiments. It can be seen that the difference in data distribution between different sessions of the same subject is much less than that between different subjects. Intuitively, this is reasonable because the difference in EEG signals between subjects is usually large due to individual factors. In contrast, the data distribution for the same subject varied less over a shorter period of time (approximately a week or longer in the SEED dataset).

Then, the same observations as the cross-subject experiment can be obtained; that is, deep learning-based methods perform better than those without deep learning, and transfer learning-based methods (such as MSJDA and JAN) outperform methods (MLP) without transfer techniques. It can be seen that the combination of transfer learning and deep learning can learn more transferable feature representations, thereby improving the effect of knowledge transfer. Likewise, JDA matching the joint distribution clearly outperforms TCA matching the marginal distribution in terms of average accuracy.

Additionally, we observe that SVM performed surprisingly well, even better than the transfer learning methods TCA and JDA. There may be two reasons for this. On the one hand, the data distribution of different sessions of the same subject does not vary greatly. On the other hand, SVM covers all source samples, while TCA and JDA only use a subset of the source samples due to limited memory. Under the influence of these factors, the transferability of shallow transfer learning methods is limited.

Finally, our proposed MSJDA achieved the best results, improving by 1.96% (*p* < 0.01) and 2.62% (*p* < 0.001) over JAN and MEERNet, respectively. In connection with the previous cross-subject experiments, it can be seen that when there is a large difference across domains and the number of source domains is large, MSJDA can achieve a more significant performance improvement compared to the baseline method. When the number of source domains increases, the framework of MSJDA can be easily extended. This makes a lot of sense in practical applications when we have data from multiple domains with different distributions.

## Discussion

### Computational cost

**FIGURE 9:**
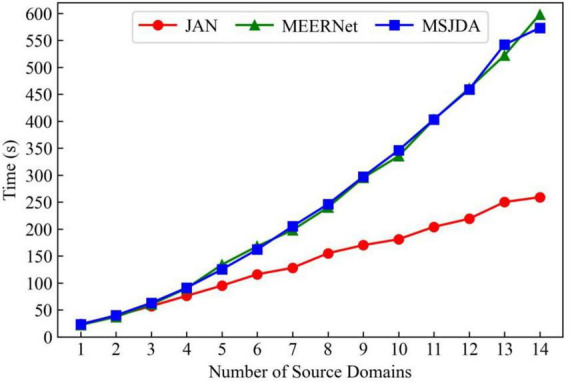
Convergence times of JAN, MEERNet and MSJDA.

The domain-shared feature extractor of MSJDA and MEERNet has the same amount of network parameters as that of JAN, while the domain-private feature extractors and label predictors of MSJDA and MEERNet have *K* times the amount of network parameters of JAN, where *K* is the number of source domains. We compare the computational cost of JAN, MEERNet, and MSJDA by comparing their convergence times in a single cross-subject transfer experiment. As shown in Figure 9, as the number of source domains increases, JAN has an advantage in the convergence time, while the convergence times of MEERNet and MSJDA are very close. This means that MSJDA achieves better transfer results for EEG emotion recognition at a very close computational cost to MEERNet.

### Confusion matrix

**FIGURE 10:**
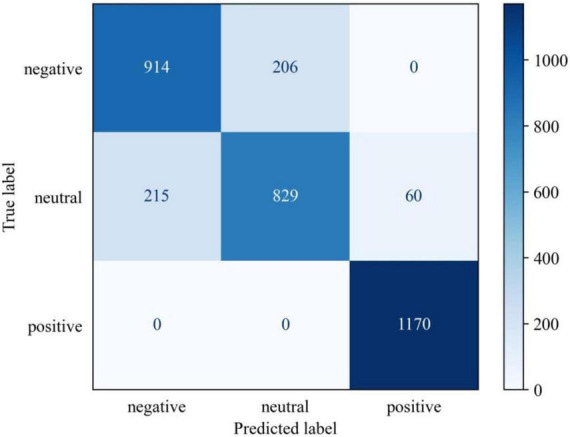
The confusion matrix of predictions in a cross-subject transfer experiment.

The confusion matrix of predictions for MSJDA in a cross-subject transfer experiment is shown in Figure 10. It can be found that the prediction accuracy of positive emotion is the highest, followed by negative emotion and neutral emotion. Additionally, negative emotion is easily predicted as neutral emotion and vice versa. This indicates that negative emotion may be close to neutral emotion, making it difficult to distinguish one from the other.

### Superiority

Based on the previous experimental results and findings, below we will discuss the reasons why MSJDA outperforms the other methods. (1) MSJDA is constructed by a neural network, which can extract more transferable features compared with shallow methods (such as TCA and JDA). (2) MSJDA aligns the joint distribution of features and predictions in the network, which can make the source and target domains more similar than the baseline method, MEERNet that only aligns the marginal distribution. (3) Compared to the baseline methods (such as JAN) that only consider a single source domain, MSJDA is able to exploit multiple available source domains to reduce the distribution difference between each source domain and target domain, thus achieving better performance with the help of multiple label predictors. (4) MSJDA benefits from being able to integrate all these advantages. In other words, MSJDA has the ability to embed a joint adaptation module into the neural network and utilize each of the multiple source domains for adaptation with the target domain separately. Therefore, MSJDA outperforms the baseline methods in both cross-subject and cross-session EEG emotion recognition.

## Conclusion

In this paper, we address the challenge of the insufficient generalization abilities of the EEG emotion recognition models in cross-subject and cross-session scenarios. To better transfer knowledge from multiple source domains to the target domain, we propose a multi-source joint domain adaptation network that separately matches the joint distributions of deep features and classification predictions for each pair of source and target domains. The cross-domain EEG emotion recognition experiments demonstrate the effectiveness of the proposed method.

## Data availability statement

Publicly available datasets were analyzed in this study. These data can be found here: https://bcmi.sjtu.edu.cn/~seed/index.html.

## Author contributions

SL, LS, YF, and LW contributed to the conception and design of the study. LW organized the database. YF performed the statistical analysis. SL wrote the first draft of the manuscript. All authors contributed to manuscript revision, read, and approved the submitted version.
